# Does size matter? Examining the drivers of mammalian vocalizations

**DOI:** 10.1111/evo.13128

**Published:** 2016-12-13

**Authors:** Kobe Martin, Marlee A. Tucker, Tracey L. Rogers

**Affiliations:** ^1^ Evolution and Ecology Research Centre, School of Biological, Earth and Environmental SciencesUniversity of New South WalesSydneyAustralia; ^2^ Senckenberg Biodiversity and Climate Research Centre and Department of Biological SciencesGoethe University FrankfurtGermany

**Keywords:** Acoustic adaptation hypothesis, acoustic behavior, body size, evolutionary allometry, frequency scaling rule, macroevolution, phylogenetic comparative analysis

## Abstract

Previous studies of the vocalization frequencies of mammals have suggested that it is either body mass or environment that drives these frequencies. Using 193 species across the globe from the terrestrial and aquatic environments and a model selection approach, we identified that the best‐supported model for minimum and maximum frequencies for vocalization included both body mass and environment. The minimum frequencies of vocalizations of species from all environments retained the influence of body mass. For maximum frequency however, aquatic species are released from such a trend with body mass having little constraint on frequencies. Surprisingly, phylogeny did not have a strong impact on the evolution of the maximum frequency of mammal vocalizations, largely due to the pinniped species divergence of frequency from their carnivoran relatives. We demonstrate that the divergence of signal frequencies in mammals has arisen from the need to adapt to their environment.

The movement of mammals back into the water over 45 million years ago has prompted many questions from evolutionary biologists. Perhaps the most important of which is, what changes occurred with this drastic change in environment? Most comparative studies of mammalian acoustic evolution focus on either terrestrial or aquatic species. Thus far there has been no comparison of mammals as a whole to determine if this monumental change has driven acoustic communication in as yet undefined ways.

Acoustic signals are critical to the survival success of many species, particularly when it comes to communication, breeding, migration, and location of prey. There is long standing debate over the evolutionary drivers of acoustic signals in mammals, with two dominant theories; the physical constraint of body size, or the environment that an animal lives within, that has driven the divergence of vocalization frequencies.

One theory that has been extensively explored is that body size, or body mass, is a driver of the evolution of acoustic signaling in mammals (Fitch 2000; Reby and McComb 2003; Fletcher 2004), as the larger an animal's size, the lower the vocalization frequency it can produce. The effect of body mass on vocalizations has been studied in the form of body mass‐frequency allometry, and has long been argued to be the main evolutionary driver behind acoustic signals (Ryan and Brenowitz 1985; Laiolo and Rolando 2003). This is denoted by the body scaling rule originally proposed by Bradbury and Vehrenkamp (1998):

(1) $$f\propto M^{-0.33}.$$

(Bradbury and Vehrenkamp 1998)

Where *f* is frequency and *M* is body mass. This relationship arises because body size limits the size of an animal's sound producing organs such as the length of the vocal tract, affecting vocalization frequencies and the formants (Ryan and Brenowitz 1985; Peters and Peters 2010).

A second hypothesis, which rivals the body size theory, argues that the environment is the dominant evolutionary driver of vocalization abilities in mammals (Morton 1975; Wiley and Richards 1978; Kime et al. 2000; Saunders and Slotow 2004; Peters and Peters 2010). The acoustic adaptation hypothesis (AAH) explains that within an environment there are many objects that obstruct the path of signals between communicating animals (Morton 1975). These differences within a species’ environment, such as different densities of vegetation, can cause a species to adopt a different vocalization frequency to optimize its success (Peters and Peters 2010). It has been proposed that to maximize communication distance, mammals would use an optimal frequency that accounts for absorption in air (Fletcher 2004) derived as:

(2) $$f\propto M^{-0.4}.$$

(Fletcher 2004)

The movement of mammals back to the water from the terrestrial environment is of great interest in terms of evolutionary change. The issue of acoustic coupling in the water, and the increase in pressure has resulted in aquatic and semiaquatic species evolving a number of strategies, residual modifications from diving adaptations to cope with the change in pressure and retention of oxygen (Reidenberg and Laitman 2010), to effectively produce sound underwater (Fig. 1). Sound absorption in water is less than that in air, and it is therefore likely that the optimal frequency needed to maximize communication distance would be altered. This shift in frequency can be predicted by:

(3) $$f\propto M^{-0.6}.$$

**Figure 1:**
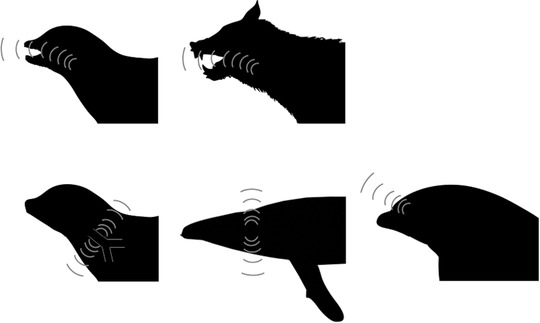
Illustration of vocalization strategies of mammals in‐air and underwater. (i) Pinnipeds in‐air vocalization through the larynx; (ii) Pinnipeds underwater vocalization potentially via expansion of the tracheal membrane; (iii) Terrestrial species’ in‐air production via perpendicular vocal folds in the larynx; (iv) Mysticete underwater production of sound using parallel vocal folds in the larynx; (v) Odontocete underwater production via phonic lips and the melon.Silhouettes by Tracy Heath, Steven Traver, and Chris Huh were downloaded from http://phylopic.org.

(Fletcher 2004)

In addition aquatic mammals have some of the greatest body masses, up to 150,000 kg, allowing us to see how the transition and subsequent increase in body mass has influenced vocalizations.

While environment and body mass as potential drivers are well established and highly successful hypotheses, there are a number of less‐developed hypotheses that have arisen recently. As vocalization has been said to function in the maintenance of group cohesion, sociality has been explored in a number of mammalian species, largely from the aquatic environment (May‐Collado et al. 2007) but also in nonhuman primates (Ramsier et al. 2012). It is suggested that species that live in larger groups have higher minimum frequencies of vocalizations, whereas nonsocial species have lower minimum frequencies due to the increased distances over which they communicate (May‐Collado et al. 2007). Low frequency sound waves are characterized by longer wavelengths than those of high frequency, and can travel greater distances (Richardson et al. 1995) with less absorption or scattering by particles as they travel through the environment, and thus would be more suitable for use by nonsocial species and species that are widely distributed. In addition, high frequencies are typical of alarm calls, allowing them to be heard over low‐frequency ambient noise. Since larger social group size is often used to deter predators, larger groups would find cause to utilize these predator‐specific alarm calls more frequently, resulting in a relationship between the increase in social group size and high‐frequency vocalizations (Ramsier et al. 2012). The Dolphin Hypothesis (Herman and Tavolga 1980) also suggests that high‐frequency whistles evolved in concert with sociality in the delphinids, however whistles have also been found in other groups of nonsocial aquatic mammalian species.

We re‐examined the possible drivers of vocalizations in mammals. Recent technological advancement has seen an increase in the amount of data collected on aquatic mammals’ vocalizations, allowing for a more robust and comprehensive comparison of terrestrial and aquatic species. Using minimum and maximum vocalization frequencies obtained from the literature, we investigated the strength of potential drivers in the evolution of mammalian vocalization. Using species from across a broad group of over 190 mammalian species we re‐examined the influence of environment and body mass and also consider the more novel driver of sociality. As body size is a known dominant driver of terrestrial vocalization we considered body size to be a fixed component in all our models. We propose three hypotheses based on the drivers proposed: (1) mass: a negative relationship between body mass and frequency, such that larger species produce lower frequencies; (2) environment: aquatic mammals will produce lower frequencies than terrestrial species to account for the larger distances over which they communicate, and semiaquatic species will be intermediate between the two; (3) and sociality: social species will produce higher frequencies due to their close proximity to conspecifics and solitary species will use lower frequencies as they degrade less quickly and are able to propagate further.

## Methods

#### Database

A database was collated of vocalization data (minimum and maximum frequency), measured in kilohertz (kHz), for all available mammalian species from the literature (Supporting Information S1). Searches were carried out using the Web of Science, Scopus, and Google Scholar databases. Searches included variations of the terms: acoustic, acoustic repertoire, mammal vocalization/vocalization, vocal, vocal communication, vocal repertoire. The majority of results were repertoire studies; however some were studies of a single call type (21 studies of single call type and these were all contact calls). The full dataset is available on the Dryad data repository (https://doi.org/10.5061/dryad.289kh).

We examined all literature to obtain the complete vocal repertoire for each species from both sonograms and tabulated data. From the vocal repertoire we identified the signal with the lowest, and the signal with the highest peak frequency. Using peak frequency resulted in the exclusion of any lower or higher energy components (formants, higher harmonics etc.), reducing the error associated with selective frequency shift. Frequency shift can result from: (1) variable distance to the calling animal from the receiving system. A product of this is the loss of high frequency components due to propagation loss, such as in dense vegetation, distortion of the signal etc.; (2) recording quality due to the frequency response limitations of the receiving system for example clipping low or high frequency components; and (3) the settings used for the analysis in the production of sonograms. The peak frequency remains unchanged. By minimising these sources of error it is unlikely that they will add bias to our results. For many species there was insufficient data to identify the behavioral context in which the signals are produced, particularly in forest dwelling primate species and aquatic species. Only vocalizations produced by adults were included as juveniles are known to produce higher frequencies. Both males and females were included. The majority of studies reported on vocalizations from both genders; however of those that reported the gender of the animals, only 10 studies focused on a single gender, usually male. Vocalization minimum frequency consisted of 170 species and maximum frequency 189 species.

The traits of body mass, environment (physical habitat), and sociality were collected for all mammalian species in the database. Body mass data was obtained from the PanTHERIA database (Jones et al. 2009), and from the published literature. Environment was categorized as one of three categorical variables (terrestrial, semiaquatic, and aquatic), grouping all habitat types into one of these broad inclusive categories. Aquatic mammals were defined as a species that relies upon an aquatic environment for any combination of breeding, locomotion, and/or feeding, and those species that rely on both the water and the land were categorized as “semiaquatic.” Sociality of species was defined to be either solitary or social, where social species lived in groups of three or more, such that solitary mother–offspring and mating pairs that were not part of a larger colony or group were not considered social. Sociality was determined from social group size and population density data from the PanTHERIA database, and also from the literature.

#### Phylogeny construction

The species for each of the two frequency limits were compiled into phylogenetic trees to be used in a phylogenetic generalized least squares (PGLS) analysis, to account for phylogenetic relatedness that could confound variation in vocalization frequencies (Laiolo and Rolando 2003). The mammalian supertree (Faurby and Svenning 2015) was pruned in R ver. 3.0.1 to include only the species for which data was available for each frequency limit. This tree included one thousand iterations to resolve any polytomies that may have been present. A supplementary analysis was carried out using a further pruned tree that excluded species from the original supertree that had had their time of divergence interpolated. This resulted in a tree containing 159 and 174 species for minimum and maximum frequency, respectively.

#### Analysis

A model selection approach was applied to test for the suitability of models to explain the evolution of the two vocalization frequency limits. Stepwise regression was carried out using backward elimination in the MASS package in R (ver 3.0.1) starting from an additive model of all variables, to determine which variable to drop in successive models. The models tested compared vocalization limits (minimum frequency and maximum frequency) with the following models: a Null model (β_0_), a Body Mass model to show the effect of body mass on frequency $\left(\beta_{0}+\beta_{mass}\right)$, two Environment models, one to test for different slopes of environments $\left(\beta_{0}+\beta_{mass}*\beta_{environment}\right)$., and one to test for a uniform slope with different intercepts, used to determine the contribution of environment $\left(\beta_{0}+\beta_{mass}+\beta_{environment}\right)$., and an additive model with all three variables $\left(\beta_{0}+\beta_{mass}+\beta_{environment}+\beta_{sociality}\right)$. A Sociality model was used to calculate the contribution of sociality $\left(\beta_{0}+\beta_{mass}+\beta_{sociality}\right)$. The caper package in R ver. 3.0.1 was used to carry out PGLS analyses and calculate Akaike's Information Criterion (with a correction for sample size; *AICc*) for each model.

The model with the lowest *AICc* is representative of the model with the highest support, though models within two units (∆*AICc_i_* < 2) of the lowest model are also considered to have sstantial evidence (Mazerolle 2004). Akaike weights were calculated using the formula:

(4) $$w_{i}\left(AICc\right)=\;\frac{\exp\left\{-\frac{1}{2}\Delta_{i}\left(AICc\right)\right\}}{\sum_{k=1}^{k}\exp\left\{-\frac{1}{2}\Delta_{k}\left(AICc\right)\right\}}.$$

(Akaike 1978; Wagenmakers and Farrell 2004)

Where *i* is the candidate model and *k* represents all the plausible candidate models. The conditional probability that the model with the lowest AICc is more likely to be the best model was also calculated using the formula:

(5) $$\frac{w_{i}\left(AICc\right)}{w_{j}\left(AICc\right)}.$$

(Wagenmakers and Farrell 2004)

Where *i* is the best fitting model and *j* is the candidate model being compared. In our case this was the second best fitting model.

The PGLS analysis also produced the parameter lambda (λ), representing the influence of phylogeny on the accumulation of changes along branches over time. λ approaching 0 indicates variation in the trait being studied is less similar between species than would be expected from their relatedness and therefore independent of phylogeny, whereas λ approaching 1 assumes these accumulated changes are linked to phylogenetic relatedness, known as a Brownian Motion model of evolution (Pagel 1999; Freckleton et al. 2002).

## Results

#### Minimum frequency

The Mass + Environment model had the lowest *AICc* value (Table 1), making it the best supported model for describing the driver of the minimum frequency of vocalizations. The model accounted for 33% of variance (*r* = 0.57). From Akaike weights the Mass + Environment model was shown to be 2.9 times more likely to be the best model than the next ranked model. An Akaike weight of 0.66 suggested little model selection uncertainty. In addition, the remaining models had ∆*AICc* values > 2, thus proving less likely to be the driver of minimum vocalization frequency. Contributions were determined using *r*‐squared values from the additive models. Mass had the highest contribution at 18% (*r* = 0.42), with a 15% contribution by environment (*r* = 0.57), and <1% by sociality (*r* = 0.42). λ approached the upper limit for the Mass + Environment model, which suggests a Brownian Motion model of evolution.

**Table 1:** A comparison of the level of support for possible explanatory models that describe the evolution of the minimum frequency in vocalizations of mammals

			95% CI of slope parameter		λ 95% CI	
Model	∆*AICc*	Weighted AICc	(lower, upper)	PGLS λ	(lower, upper)	Effect size (*r*)
β_0_ + β_mass_ + β_environment_	0.00	0.6644	–0.50, –0.32	0.53	0.53, 0.54	0.57
β_0_ + β_mass_ + β_environment_ + β_sociality_	2.13	0.2290	–0.50, –0.32	0.53	0.53, 0.54	0.57
			–0.18, 0.25 (T)			
β_0_ + β_mass*_β_environment_	4.09	0.0860	–0.34, 0.51 (S)	0.51	0.50, 0.51	0.59
			–0.63, –0.26 (A)			
β_0_ + β_mass_	7.56	0.0152	–0.42, –0.21	0.79	0.79, 0.79	0.42
β_0_ + β_mass_ + β_sociality_	9.59	0.0055	–0.42, –0.21	0.79	0.79, 0.79	0.42
β_0_	33.98	0.0000	–	0.89	0.89, 0.90	–

T is terrestrial, S is semiaquatic, and A is aquatic.

The results are produced from phylogenetic generalized least squares (PGLS) analysis.

**Figure 2:**
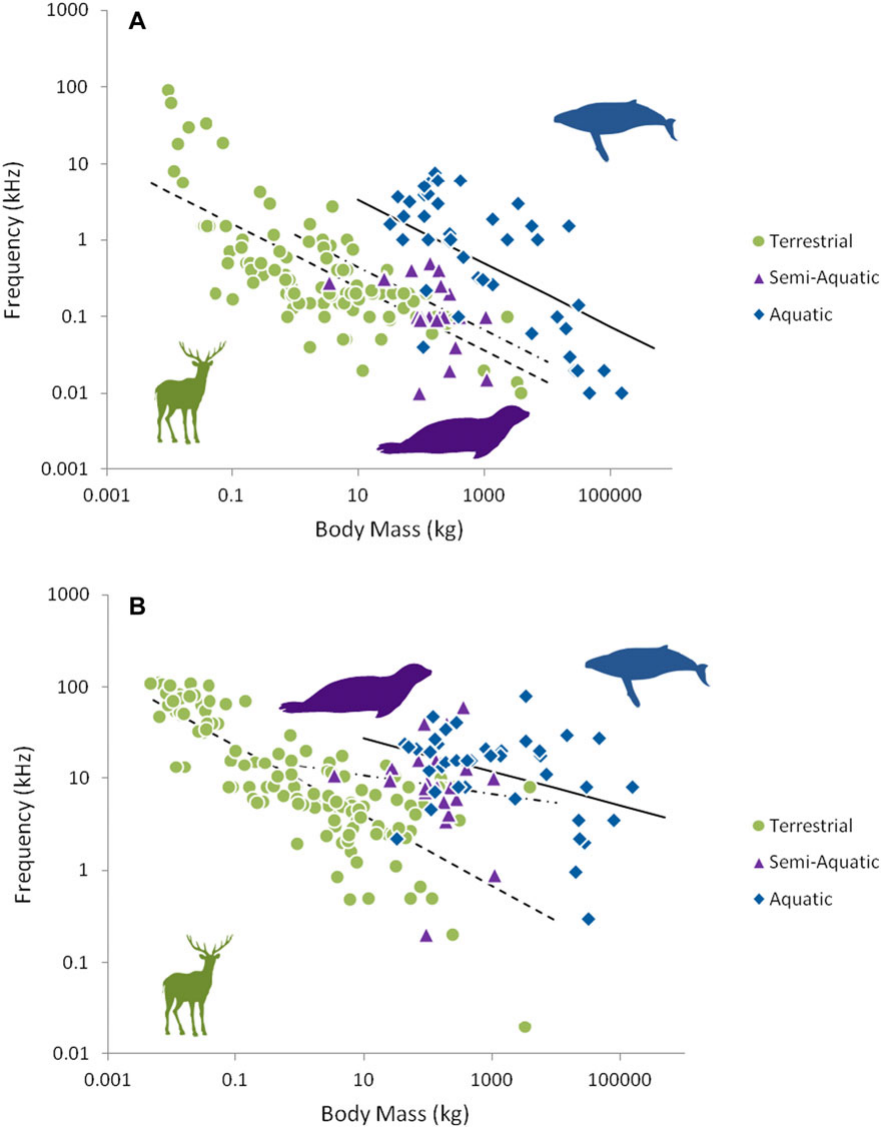
(A) Vocalization minimum frequency as a function of species body mass for terrestrial (*n* = 105), semiaquatic (*n* = 23), and aquatic (*n* = 42) environments on a log scale. The dotted line represents the phylogenetic generalized least squares (PGLS) regression line for terrestrial mammals log(Y) = –0.41log(X)–0.21 (CI –0.50, –0.32) the dash‐dot line represents semiaquatic mammals log(Y) = –0.41log(X) + 0.06 (CI –0.50, –0.32) and the solid line for aquatic mammals log(Y) = –0.41log(X) + 0.93 (CI –0.50, –0.32). (B) Vocalization maximum frequency as a function of species body mass for terrestrial (*n* = 125), semiaquatic (*n* = 23), and aquatic (*n* = 41) environments on a log scale. Terrestrial mammals log(Y) = –0.38log(X) + 0.98 (CI –0.34, –0.06), semiaquatic mammals log(Y) = –0.18log(X) + 1.13 (CI –0.28, 0.45), and aquatic mammals log(Y) = –0.18log(X) + 1.63 (CI –0.31, –0.06). Silhouettes by Oscar Sanisidro and Chris Huh were downloaded from http://phylopic.org.

Terrestrial, semiaquatic and aquatic mammals have the same trend (slope = –0.41 ± 0.05) of minimum frequency in vocalizations by body mass (Fig. 2A). Mammals in the aquatic environment have on average higher minimum frequencies (intercept = 0.93) in their vocalizations than semiaquatic species (intercept = 0.06), which in turn are higher than those of terrestrial mammals (intercept = –0.21).

#### Maximum frequency

Mass × Environment was the best supported model, calculated from Akaike weights to be 10.5 times more likely to be the driver of maximum frequency in the vocalizations of mammals (Table 2) than the next ranking model, and accounting for 53% of variance (*r* = 0.73). An Akaike weight of 0.88 suggested substantial model selection certainty. All other candidate models possessed ∆*AICc* values > 2 and Akaike weights <0.1, suggesting these models are unlikely to drive the differences in high vocalization frequency thresholds among mammals, as was supported by their Akaike's weights (Table 2). Environment contributed 25% (*r* = 0.44), mass contributed 8% (*r* = 0.29), and sociality 1% (*r* = 0.30). The λ value of the Mass × Environment model was on the lower bound of 0 suggesting the observed values evolved independently of phylogeny.

**Table 2:** A comparison of the level of support for possible explanatory models that describe the evolution of maximum frequency limits of vocalizations

			95% CI of slope parameter		λ 95% CI	
Model	∆*AICc*	Weighted AICc	(lower, upper)	PGLS λ	(lower, upper)	Effect size (*r*)
			–0.34, –0.06 (T)			
β_0_ + β_mass*_β_environment_	0.0	0.8846	–0.28, 0.45 (S)	0	0, 0	0.73
			–0.31, –0.06 (A)			
β_0_ + β_mass_ + β_environment_	4.7	0.0844	–0.36, –0.24	0.23	0.23, 0.23	0.58
β_0_ + β_mass_ + β_environment_ + β _sociality_	6.7	0.0310	–0.36, –0.24	0.24	0.24, 0.24	0.57
β_0_ + β_mass_	26.05	0.0000	–0.24, –0.08	0.66	0.66, 0.67	0.29
β_0_ + β_mass_ + β_sociality_	26.58	0.0000	–0.24, –0.08	0.68	0.67, 0.68	0.30
β_0_	37.89	0.0000	–	0.77	0.77, 0.77	–

T is terrestrial, S is semiaquatic, and A is aquatic.

Results are produced from phylogenetic generalized least squares (PGLS) analysis.

Terrestrial, semi‐aquatic, and aquatic mammals have very different trends of maximum frequencies in their vocalizations as a function of body mass (Fig. 2B). Terrestrial species have a stronger relationship (slope = –0.38 ± 0.07, intercept = 0.98) between frequency and mass than semi‐aquatic (slope = –0.18 ± 0.19, intercept = 1.13) and aquatic species (slope = –0.18 ± 0.06, intercept = 1.63).

#### Removal of Interpolated species

The removal of interpolated species from the composite tree resulted in similar lambda values for all models for minimum frequency (Supporting Information S2). For maximum frequency, the removal of these species resulted in a larger lambda value for the top three models, while the others remained similar (Supporting Information S2).

## Discussion

This is the first study to examine and compare the evolutionary drivers of the vocalizations of mammals of both terrestrial and aquatic species. For both minimum and maximum frequency limits of vocalization, body mass, and environment together consistently best described the evolution of vocalization in mammals, performing better than either body mass or environment alone. Our results demonstrate that both body mass and environment contributed in varying degrees to the evolution of vocalizations.

Mammals returned to the water from the land in five extant groups belonging to three orders (Carnivora, Cetartiodactyla, and Sirenia). In‐air vocalizers face different challenges than those communicating underwater. Terrestrial species must overcome interference caused by vegetation density, weather, temperature barriers etc., while aquatic species compete with inhomogeneities such as salinity and temperature discontinuities, underwater landscapes, and bathymetry (Urick 1982). On top of these factors, sound travels differently through air and water, and these differences are taken into account when adjusting to the challenges faced in the communication medium, to maximize the success of signal transmission. Aquatic mammals have shifted to use higher frequency vocalizations compared to terrestrial mammals of similar body mass. This is most likely due to the beneficial propagation properties of water.

#### Body Mass

**Figure 3:**
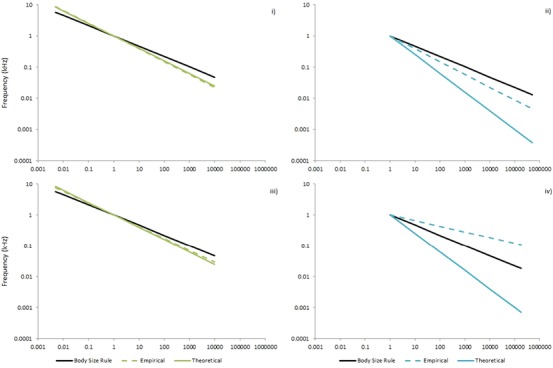
Comparison of regressions based on the Body Size Rule, theoretical equations based on those by Fletcher (2004), and empirical data for (i) terrestrial minimum frequency, (ii) aquatic minimum frequency, (iii) terrestrial maximum frequency, and (iv) aquatic maximum frequency limits of vocalizations.

We found that the minimum frequency of vocalizations of mammals in all three environments retained the negative relationship with body mass; with the minimum frequency limits of vocalizations of aquatic mammals being higher in frequency than those of terrestrial mammals of a similar body mass. The resulting slope (–0.41) is more similar to the –0.4 slope proposed by Fletcher (2004) that takes into account environmental factors, than the slope predicted by the body scaling rule (–0.33) proposed by Bradbury and Vehrenkamp (1998) (Fig. 3i).

As was the case for the minimum frequency limits of vocalizations, mammals of the aquatic environment demonstrated higher maximum frequency limits. Our results confirmed the negative relationship between body mass and maximum frequencies in vocalization for terrestrial and aquatic species, though this was less pronounced in the aquatic species. However, linear regression analysis found this relationship was not significant for the semiaquatic species. Therefore, while terrestrial species still retain the influence of body mass, semiaquatic, and aquatic species are not as restricted in their maximum frequency limits of vocalization. Terrestrial species had a slope (–0.38) similar to that predicted by Fletcher (2004) (–0.4), while aquatic species presented a slope (–0.18) vastly different from that proposed by both Fletcher (2004) (–0.6) and the body scaling rule (Fig. 3iii, iv). Our results show that the incorporation of allowances for a species’ environment into the preexisting body mass models is an appropriate approach for minimum and maximum frequency limits for terrestrial mammal species. In the case of aquatic and semiaquatic species environment has an even greater influence on the frequencies produced than previously predicted. It is also evident that body mass does not have as powerful an influence on mammals from these environments as was previously believed.

The retention of the relationship between body mass and minimum frequency limit was expected (Fletcher 2004; Peters and Peters 2010). To produce low minimum frequency vocalizations animals require a sound‐production system proportional in size to produce the signal. Correlations have been found between body size (mass) and the length of the vocal tract trachea as it influences the formant frequencies of the vocalizations (Reby and McComb 2003), as well as the skull morphology and palate length (Fitch 2000). Therefore large mammals have large vocalising organs and can produce lower frequency vocalizations than smaller animals (Huang et al. 2000). This relationship holds true for the minimum frequencies of mammals from all three environments and supports the findings of previous studies (Hauser 1993; May‐Collado et al. 2007). However, for the maximum frequency limits of vocalizations body mass is a weaker predictor, attributing just 8% of variance, down from 18% for minimum frequency. This is not unexpected. While body size is a limiting factor for minimum frequencies, the same size relationships do not apply for producing maximum frequencies and mammals have developed other strategies for producing higher frequency sounds, which can be implemented by a relatively small area or apparatus in relation to body size. For example, the superfast‐moving laryngeal muscles of echolocating bats (Elemans et al. 2011), and the production of high‐frequencies through a system involving phonic lips and the melon in some odontocetes (toothed whales) (Au and Hastings 2008).

#### Environment

Our results newly highlight the importance of environment in driving the frequency limits of vocalizations in mammals. The concurrence of our empirical slopes with the theoretical slopes provided by Fletcher (2004) for terrestrial species offers support to our conclusion that it is an important combination of both body mass and environment that drive these frequency limits (Fig. 3i, iii). However, it is the complete deviation of aquatic species from either the body mass rule (Bradbury and Vehrenkamp 1998) or the environment corrected slope (Fletcher 2004) that accentuates the power of a species’ environment in driving their vocalization frequency limits (Fig. 3ii, iv).

As a general pattern aquatic and semiaquatic mammals use higher frequency vocalizations than terrestrial mammals both at the minimum and maximum limits of their repertoires. This resulted in species of similar body mass producing frequencies some 10 kHz apart. For example, in the terrestrial environment *Panthera tigris* weighs in at 162 kg and has a frequency range of 0.1–10 kHz, whereas the aquatic *Stenella coeruleoalba* has a mean mass of 142 kg and produces frequencies of 6–24 kHz. Sound travels faster through water than through air (∼ five times faster), although propagation speed and distance are affected by local characteristics such as bathymetry, substrate, and the presence of boundary layers (Urick 1982; Au and Hastings 2008). This means that wavelengths of sound traveling through water are shorter than expected (Madsen and Surlykke 2013). The result is that higher frequency sounds can travel further through water in the aquatic environment with less loss of acoustic energy than they would be able to in the terrestrial environment (Forrest 1994). This phenomenon is accentuated in the Arctic and Antarctic regions, where the water column below the surface is comprised of an isothermal layer that normally occurs at deep abyssal depths, and sound velocity is increased (Urick 1982). This means higher frequency sounds can travel faster and therefor further in the waters near the surface where many pinniped and cetacean species spend much of their time. The loss of acoustic energy due to absorption increases with the frequency of the call and this loss occurs at a lower level in water than in air. Thus, the aquatic species are able to utilize higher frequencies than the terrestrial species of similar body mass are.

**Figure 4:**
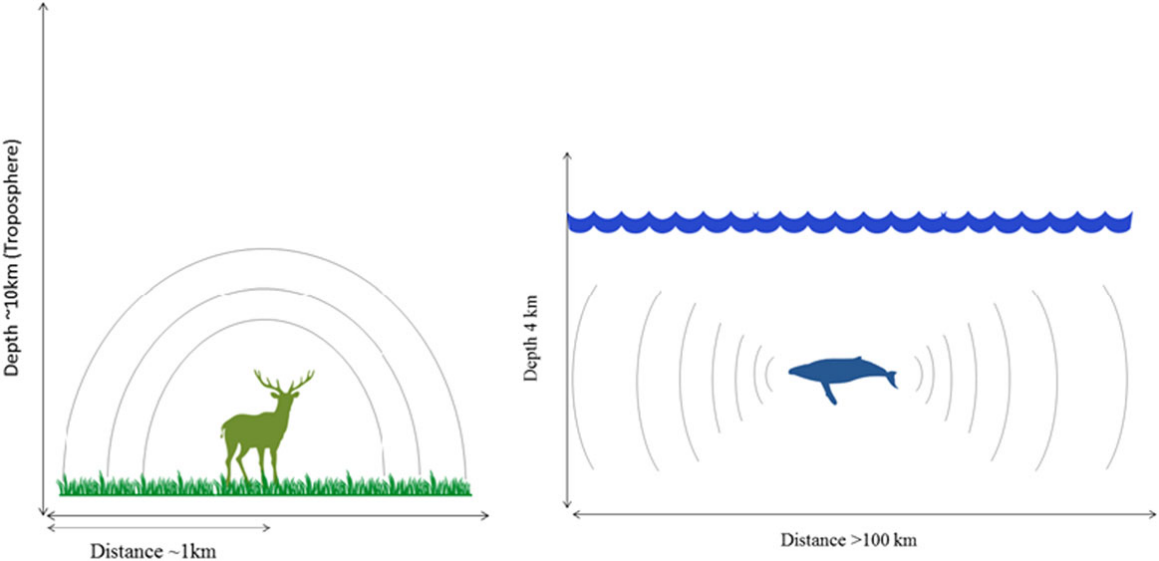
Illustration of the 2D and 3D propagation properties of the terrestrial and aquatic environments, respectively. Silhouettes by Oscar Sanisidro and Chris Huh were downloaded from http://phylopic.org.

In terrestrial landscapes vocalizations behave in a 3‐dimensional pattern of propagation (Fig. 4). The troposphere extends to approximately 10 km above ground level, while a terrestrial mammal call generally only travels up to 1 km from the source, with elephant calls traveling as far as 2.5 km (McComb et al. 2003). With the depth of the environment extending further than the distance of a call, the sound propagates in a spherical manner. Sound propagation in the aquatic environment however, takes on a 2‐dimensional cylindrical property (Fletcher 2004) (Fig. 4). Ocean depth is 4 km on average, with most aquatic mammal species occupying areas of 1 km depth or less. The vocalizations of aquatic species therefore travel greater distances, up to 1000 km (blue whale), than the depth of their environment, and hence propagate in a cylindrical manner (Richardson et al. 1995). Most vocalizations are not able to propagate all the way to the ocean floor due to temperature and salinity profiles within the water column. A surface duct is often present in the layer just below the ocean surface where sound is “trapped,” bound by the ocean surface and the lower boundary of the duct in a cylindrical propagation path (Urick 1982). Sound attenuates more rapidly with distance with spherical (terrestrial environments) rather than cylindrical (aquatic environments) spreading (Richardson et al. 1995; Fletcher 2004), hence the increased propagation of sounds and higher frequencies produced by species vocalizing in the aquatic environment compared with the terrestrial environment.

**Figure 5:**
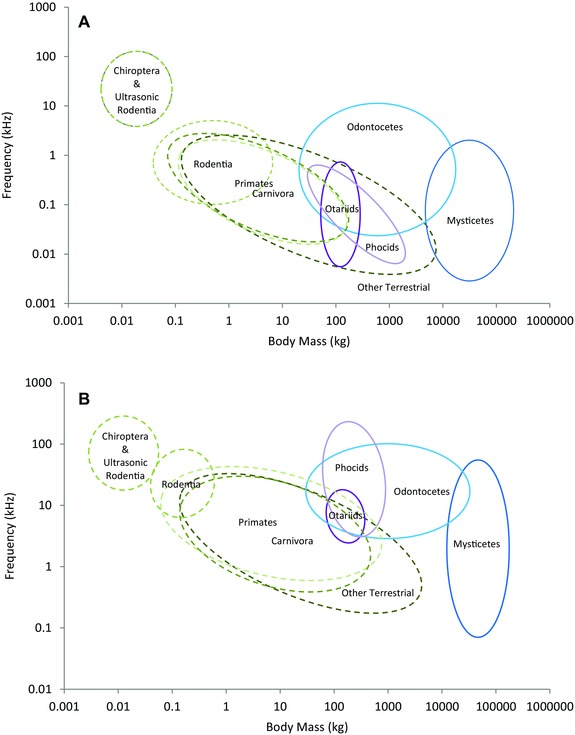
A graphical summary of vocalization (A) minimum and (B) maximum frequency showing general trends for functional groups of acoustic interest.

It is possible that the demarcation resulting from the shift in semiaquatic species is responsible for the boost in the contribution of the environment model. It may also be responsible for the decrease in influence from phylogeny. For minimum frequency, pinnipeds were situated with their terrestrial cousins in the Carnivora, whereas they produce more similar maximum frequencies to the true aquatic species, such that the influence of environment is stronger than that of phylogeny. Pinnipeds, as an amphibious group, require a communication system that is effective both in air and underwater. The pinnipeds were situated within the terrestrial data points and for the majority, fit to the terrestrial trend line (Fig. 5A). The three aquatic otter species are similarly nested within the terrestrial data points. These species vocalize in air in a similar manner to terrestrial species. This fits with the amphibious lifestyle of these two groups, inhabiting both the land and water. For the pinnipeds, there was no discernible difference between the frequencies produced by phocids (true seals), of which most species communicate underwater, and otariids (eared seals), generally in‐air communicators. These groups appear to act as a functional intermediate between the terrestrial and true aquatic species at low frequencies.

Where the pinnipeds and aquatic mustelids were positioned more with terrestrial mammals for their minimum frequencies, their maximum frequencies told a very different story. The otariids remained close to the terrestrial species, while the phocids showed much higher frequencies, clearly breaking away from the terrestrial species and displaying frequencies similar to the odontocetes (Fig. 5B). This begs the question, why do these two groups behave so similarly in their minimum frequencies and yet produce vastly different maximum frequencies of vocalizations? While phocids spend more time underwater than otariids, their amphibious lifestyle means that even they must return to land (Schusterman et al. 2004). Studies have shown that while pinniped ears were indeed an intermediate between terrestrial and fully aquatic ears, otariid ears were primarily air‐adapted, while phocids demonstrated an affinity to underwater hearing (Kastak and Schusterman 1998). Now examining vocalizations, a similar pattern is emerging. While it may be energetically easier to produce higher frequencies, those higher frequencies are less useful in a terrestrial environment as they attenuate and lose information more rapidly. The terrestrial‐like minimum frequencies produced by both pinniped groups are therefore likely to be a consequence of needing to transfer information over long distances and in noisy conditions while on land (colonial breeding, social aggregations, locating mates/offspring). Otariids, spending the majority of their time on land, are more constrained by the acoustic propagation properties of the terrestrial environment for their maximum frequency limits of vocalizations as well. However, the majority of phocids vocalize underwater. It is probable that they have adapted to utilize the propagation efficiency of the aquatic environment in a similar fashion to the fully aquatic cetacean species. Some Arctic (*Halichoerus grypus* (Asselin et al. 1993), *Phoca vituline* (Renouf et al. 1980), *Pagophilus groenlandicus* (Richardson et al. 1995), *Pusa hispida* (Cummings et al. 1984)), and Antarctic (*Hydrurga leptonyx* (Thomas et al. 1983b), *Leptonychotes weddellii* (Thomas and Stirling 1983)) phocid species have been shown to produce maximum frequencies in the same range as echolocating odontocetes (for example, the leopard seal is capable of producing frequencies up to 60 kHz (Thomas et al. 1983a)). While the debate as to whether pinnipeds do or do not echolocate has been mostly closed, these results suggest it may be appropriate to reexamine the acoustic behaviors of this group, particularly with an ultrasonic focus.

#### Sociality

The results of the stepwise regression showed that sociality became less important as a driver of the maximum frequencies produced by mammals. It has been suggested that the vocalization abilities of a species are in large part focused on optimizing maximum long‐distance communication (Wiley and Richards 1978; Kime et al. 2000; Fletcher 2004; Saunders and Slotow 2004; Peters and Peters 2010). Since low frequencies are characteristic of long‐distance calls, and solitary species are more likely to require their vocalizations to travel greater distances than a social species, it may be expected that there would be some difference in the degree to which species of different levels of sociality may need to exploit the long‐ranging characteristics of these frequencies.

However, not all animals use their vocalizations over the maximum distances possible. Species may employ different strategic uses of frequencies depending on the contextual demands (motivation for the use of a particular sound (Morton 1977)) and the influences of their life history. For example, social or contact calls are often used by both social and solitary species. These calls do not need to travel far and are therefore characteristically of high frequency. It would therefore not be expected that there would be a great amount of difference between the two groups’ maximum frequencies, as is portrayed in our results by the sociality variable being the first to be dropped from the additive models. It has thus been suggested that a focus should perhaps be put on features correlated with spacing between signaller and receiver (Wiley and Richards 1978; Peters and Peters 2010).

For both minimum and maximum frequency vocalization limits environment and body mass only accounted for 33% and 53% of variance, respectively, leaving a large amount of variance as yet unexplained by any of the drivers we examined, with the potential for further studies of other contributors. Previous studies (Saunders and Slotow 2004) have suggested that long‐ and short‐range communication is determined by differing ecologies (mating systems and territory size). It is possible that such ecologies are in part responsible for the remaining 50–70% of variance.

Background noise in both the terrestrial and aquatic environments has been increasing over the past few decades, largely due to anthropogenic activities. Background noise is a major contributor to degradation of sounds in the environment, which in turn can lead to inability of a receiver to recognize or receive a signal (Kime et al. 2000). Echolocating species that rely on sound for feeding and navigation are one example of a group of species highly susceptible to impacts from the increase in anthropogenic noise due to their heavy reliance on acoustic communication. As a result of a receiver's inability to distinguish calls from conspecifics amongst the background noise, the broadcaster will need to alter their vocalizations or behaviors so as to optimize transmission of the signals in their environment (Saunders and Slotow 2004).

There are some caveats involved with this type of study, for example the wide variation in the types of calls recorded (Fletcher 2004), with no dataset of a particular call type large enough for any significant comparison and analysis. To compare acoustic signals of vocalizations across mammals we were required to incorporate calls where the context of the calls was sometimes unknown. This is particularly the case for aquatic mammals where the intention of the signaller cannot be determined.

## Conclusion

This study has revealed a number of novel patterns in the vocalization abilities of mammals that have not previously been explored. Notably, our results highlight the importance of environment in combination with body mass in driving the acoustic communication of mammals. The evolution of vocalization frequencies in aquatic mammals has clearly diverged from terrestrial mammals as they returned to the water. The addition of aquatic species to comparative studies allows us to examine the effect of an extreme shift in environment and size, such as that of moving from the land to the water. The comparison of the amphibious pinnipeds to fully terrestrial and aquatic mammals has accentuated their role as an intermediary group. In addition, comparing the two groups of pinnipeds and their use of vocalizations has highlighted the difference in propagation properties of the two environments and how mammals have evolved to utilize these properties to their advantage.

Signal evolution is a complex trait that has been shaped by a variety of intrinsic (e.g., morphology or physiology) and extrinsic factors (e.g., environment). Understanding what has shaped the acoustic features of animal vocalization is important for understanding how it may change into the future in response to changing niches and roles, and shifts occurring in their environments. Studies such as this one, which highlight the difference in vocal behaviors of mammals, are important for identifying potential impacts from these changing niches and roles, and aiding in the development of measures to minimize any negative impacts.

Associate Editor: M. Friedman

Handling Editor: M. Noor

## Supporting information


**Supporting Information S1**. List of references for frequency data.

**Supporting Information S2**. A comparison of the level of support for possible explanatory models that describe the evolution of the a) minimum and b) maximum frequency in vocalisations of mammals.
