# Supplementary material for: Does size matter? Examining the drivers of mammalian vocalizations
Source: Evolution. 2016 Dec 13;71(2):249–60. doi: 10.1111/evo.13128 (PMC5324685; doi:10.1111/evo.13128)
Supplement: Supplementary file 1 — Supporting Information S1. List of references for frequency data. [file EVO-71-249-s001.docx]

**Electronic Supporting Information**

**Supporting Information S1.** List of references for frequency data.

1. Aitkin, L.M., B.M.H. Bush, and G.R. Gates. 1978. The auditory midbrain of a marsupial: The brush-tailed possum (*Trichosurus vulpecula*). *Brain Res.* **150**: 29-44.
2. Aitkin, L.M., J.E. Nelson, and R.K. Shepherd. 1994. Hearing, vocalization and the external ear of a marsupial, the northern quoll, *Dasyurus hallucatus*. *J. Comp. Neurol.* **349**: 377-388.
3. Asselin, S., M.O. Hammill, and C. Barrette. 1993. Underwater vocalizations of ice breeding grey seals. *Can. J. of Zoolog.* **71**: 2211-2219.
4. Attard, M.R., B.J. Pitcher, I. Charrier, H. Ahonen, and R.G. Harcourt. 2010. Vocal discrimination in mate guarding male Australian sea lions: familiarity breeds contempt. *Ethology* **116**: 704-712.
5. Beamish, P., and E. Mitchell. 1971. *Ultrasonic sounds recorded in the presence of a blue whale Balaenoptera musculus.* Paper presented at the Deep Sea Research and Oceanographic Abstracts.
6. Belan, I., P.N. Lehner, and T. Clark. 1978. Vocalizations of the American pine marten, Martes americana. *J. Mammal.* **59**: 871-874.
7. Berg, J.K. 1983. Vocalizations and associated behaviors of the African elephant (Loxodonta africana) in captivity. *Z. Tierpsychol.* **63**: 63-79.
8. Berryman, J.C. 1976. Guinea‐pig Vocalizations: Their Structure, Causation and Function. *Z. Tierpsychol.* **41**: 80-106.
9. Bezerra, B.M., A.S. Souto, and G. Jones. 2010. Vocal repertoire of golden-backed uakaris (Cacajao melanocephalus): call structure and context. *Int. J. Primatol.* **31**: 759-778.
10. Bezerra, B.M., A.S. Souto, N. Schiel, and G. Jones. 2011. Notes on vocalisations of giant otters in the flooded Igapó forests of Jaú National Park, Amazonas, Brazil. *J. Ethol.* **29**: 169-175.
11. Binz, H., and E. Zimmermann. 1989. The vocal repertoire of adult tree shrews (Tupaia belangeri). *Behaviour* **109**: 142-162.
12. Brady, C.A. 1981. The vocal repertoires of the bush dog (*Speothos venaticus*), crab-eating fox (*Cerdocyon thous*), and maned wolf (*Chrysocyon brachyurus*). *Anim. Behav.* **29**: 649-669.
13. Brenner, F.J., C.P. Gaetano, S.W. Mauser, and D.L. Belowich. 1978. Body weight and social interactions as factors in determining dominance in captive eastern chipmunks Tamias striatus. *Anim. Behav.* **26**: 432-437.
14. Budde, C., and G.M. Klump. 2003. Vocal repertoire of the black rhino Diceros bicornis ssp. and possibilities of individual identification. *Z. Saugetierkd.* **68**: 42-47.
15. Caldwell, M.C., and D.D. Caldwell. 1970. Statistical evidence for individual signature whistles in the Pacific whitesided dolphin, *Lagenorhynchus obliquidens*: DTIC Document. (No. TR-9). Los Angeles County Museum CA.
16. Capranica, R.R, E. Nevo, and A.J.M. Moffat. 1974. Vocal repertoire of a subterranean rodent (Spalax). *J. Acoust. Soc. Am.* **55**: 481-481.
17. Channing, A., and D.T. Rowe‐Rowe. 1977. Vocalizations of South African mustelines. *Z. Tierpsychol.* **44**: 283-293.
18. Charlton, B.D, Z. Zhihe, and R.J. Snyder. 2009. Vocal cues to identity and relatedness in giant pandas (Ailuropoda melanoleuca). *J. Acoust. Soc. Am.* **126**: 2721-2732.
19. Charlton, B.D., W.A. Ellis, A.J. McKinnon, G.J. Cowin, J. Brumm, K. Nilsson, *et al.* 2011. Cues to body size in the formant spacing of male koala (Phascolarctos cinereus) bellows: honesty in an exaggerated trait. *J. Exp. Biol.* **214**: 3414-3422.
20. Charrier, I., and R.G. Harcourt. 2006. Individual vocal identity in mother and pup Australian sea lions (Neophoca cinerea). *J. Mammal.* **87**: 929-938.
21. Clark, C.W. 1982. The acoustic repertoire of the southern right whale, a quantitative analysis. *Anim. Behav.* **30**: 1060-1071.
22. Clark, C.W, and J.H. Johnson. 1984. The sounds of the bowhead whale, Balaena mysticetus, during the spring migrations of 1979 and 1980. *Can. J. Zoolog.* **62**: 1436-1441.
23. Cleveland, J., and C.T. Snowdon. 1982. The Complex Vocal Repertoire of the Adult Cotton‐top Tamarin (Saguinus oedipus oedipus) 1. *Z. Tierpsychol.* **58**: 231-270.
24. Collins, K.T., T.L. Rogers, J.M. Terhune, P.D. McGreevy, K.E. Wheatley, and R.G. Harcourt. 2005. Individual variation of in-air female 'pup contact' calls in Weddell seals, Leptonychotes weddellii. *Behaviour* **142**: 167-189.
25. Compton, L.A., J.A. Clarke, J. Seidensticker, and D.R. Ingrisano. 2001. Acoustic characteristics of white-nosed coati vocalizations: a test of motivation-structural rules. *J. Mammal.* **82**: 1054-1058.
26. Credner, S., H. Burda, and F. Ludescher. 1997. Acoustic communication underground: vocalization characteristics in subterranean social mole-rats (Cryptomys sp., Bathyergidae). *J. Comp. Physiol. A* **180**: 245-255. doi: 10.1007/s003590050045
27. Cummings, W.C., D.V. Holliday, and B.J. Lee. 1984. Potential impacts of man-made noise on ringed seals: vocalizations and reactions. *NOAA, Anchorage, AK*.
28. Dawbin, W.H., and D.H. Cato. 1992. Sounds of a pygmy right whale (Caperea marginata). *Aquat. Mammal Sci.* **8**: 213-219.
29. De Waal, F.B. 1988. The communicative repertoire of captive bonobos (Pan paniscus), compared to that of chimpanzees. *Behaviour* **106**: 183-251.
30. Déaux, É.C., and J.A. Clarke. 2013. Dingo (Canis lupus dingo) acoustic repertoire: form and contexts. *Behaviour* **150**: 75-101.
31. Dempster, E. 1994. Vocalisations of adult Northern quolls, Dasyurus hallucatus. *Aust. Mammal.* **17**: 43-49.
32. East, M.L., and H. Hofer. 1991. Loud calling in a female-dominated mammalian society: I. Structure and composition of whooping bouts of spotted hyaenas, Crocuta crocuta. *Anim. Behav.* **42**: 637-649.
33. Edds, P.L., D.K. Odell, and B.R. Tershy. 1993. Vocalizations of a captive juvenile and free‐ranging adult‐calf pairs of Bryde's whales, Balaenoptera edeni. *Aquat. Mammal Sci.* **9**: 269-284.
34. Eisenberg, J.F. 1976. Communication mechanisms and social integration in the black spider monkey. *Ateles fusciceps robustus*. *Sm. C. Zoolog.* **213**: 1-108.
35. Eisenberg, J.F., L.R. Collins, and C. Wemmer. 1975. Communication in the Tasmanian devil (Sarcophilus harrisii) and a survey of auditory communication in the Marsupialia. *Z. Tierpsychol.* **37**: 379-399.
36. Epple, G. 2008. Comparative studies on vocalization in marmoset monkeys (Hapalidae). *Folia primatol.* **8**: 1-40.
37. Evans, W.E., and E.S. Herald. 1970. Underwater calls of a captive Amazon manatee, Trichechus inunguis. *J. Mammal.* **51**: 820-823.
38. Farley, S.D., P.N. Lehner, T. Clark, and C. Trost. 1987. Vocalizations of the Siberian Ferret (Mustela eversmanni) and Comparisons with Other Mustelids. *J. Mammal.* **68**: 413-416. doi: 10.2307/1381487
39. Fenton, M.B., and J.H. Fullard. 1979. The influence of moth hearing on bat echolocation strategies. *J. Comp. Physiol.* **132**: 77-86.
40. Fenton, M.B. 1982. Echolocation Calls and Patterns of Hunting and Habitiat Use of Bats (Microchiroptera) from Chillagoe, North Queensland. *Aust. J. Zool.* **30**: 417-425.
41. Fish, J.F., J.L. Sumich, and G.L. Lingle. 1974. Sounds produced by the gray whale, Eschrichtius robustus. *Mar. Fish. Rev* **36**: 38-45.
42. Ford, J.K.B., and H.D. Fisher. 1978. Underwater acoustic signals of the narwhal (Monodon monoceros). *Can. J. Zoolog.* **56**: 552-560.
43. Fossey, D. 1972. Vocalizations of the mountain Gorilla (*Gorilla gorilla beringei*). *Anim. Behav.* **20**: 36-53.
44. Fourie, P.B. 1977. Acoustic communication in the rock hyrax, Procavia capensis. *Z. Tierpsychol.* **44**: 194-219.
45. Frey, R., and A. Gebler. 2003. The highly specialized vocal tract of the male Mongolian gazelle (Procapra gutturosa Pallas, 1777–Mammalia, Bovidae). *J. Anat.* **203**: 451-471.
46. Frey, R., A. Gebler, and G. Fritsch. 2006. Arctic roars–laryngeal anatomy and vocalization of the muskox (Ovibos moschatus Zimmermann, 1780, Bovidae). *J. Zool.* **268**: 433-448.
47. Frommolt, K., M.E. Goltsman, and D.W. Macdonald. 2003. Barking foxes, Alopex lagopus: field experiments in individual recognition in a territorial mammal. *Anim. Behav.* **65**: 509-518.
48. Gamba, M., and C. Giacoma. 2007. Quantitative acoustic analysis of the vocal repertoire of the crowned lemur. *Ethol. Ecol. Evol.* **19**: 323-343. doi: 10.1080/08927014.2007.9522555
49. Gautier, J. 1974. Field and laboratory studies of the vocalizations of talapoin monkeys (Miopithecus talapoin). *Behaviour* **51**: 209-273.
50. Goldingay, R.L. 1994. Loud Calls of the Yellow-Bellied Glider, Petaurus-Australis-Territorial Behavior by an Arboreal Marsupial. *Aust. J. Zool.* **42**: 279-293.
51. Gouzoules, H., and S. Gouzoules. 1989. Design features and developmental modification of pigtail macaque, *Macaca nemestrina*, agonistic screams. *Anim. Behav.* **37**: 383-401.
52. Gwilliam, J., I. Charrier, and R.G. Harcourt. 2008. Vocal identity and species recognition in male Australian sea lions, Neophoca cinerea. *J. Exp. Biol.* **211**: 2288-2295.
53. Hafner, M.S., and D.J. Hafner. 1979. Vocalizations of grasshopper mice (genus Onychomys). *J. Mammal.* **60**: 85-94.
54. Hanggi, E.B., and R.J. Schusterman. 1994. Underwater acoustic displays and individual variation in male harbour seals, Phoca vitulina. *Anim. Behav.* **48**: 1275-1283.
55. Hauser, M.D. 1991. Sources of Acoustic Variation in Rhesus Macaque (Macaca mulatta)) Vocalizations. *Ethology* **89**: 29-46.
56. Hauser, M.D. 1993. The evolution of nonhuman primate vocalizations: effects of phylogeny, body weight, and social context. *Am. Nat.* **142**: 528-542.
57. Heller, K., and O.V. Helversen. 1989. Resource partitioning of sonar frequency bands in rhinolophoid bats. *Oecologia* **80**: 178-186.
58. Henline, W.T. 2007. *Vocal repertoire of white-faced sakis (Pithecia pithecia).* Doctoral Dissertation: Eastern Kentucky University.
59. Hill, M.S.C., J.W.H. Ferguson, M.N. Bester, and G.I.H. Kerley. 2001. Preliminary comparison of calls of the hybridizing fur seals Arctocephalus tropicalis and A. gazella. *Afr. Zool.* **36**: 45-53.
60. Hohmann, G. 1989. Vocal communication of wild bonnet macaques (Macaca radiata). *Primates* **30**: 325-345.
61. Holy, T.E., and Z. Guo. 2005. Ultrasonic songs of male mice. *PLoS Biol* **3**: e386. doi: 10.1371/journal.pbio.0030386
62. Huff, J.N., and E.O. Price. 1968. Vocalizations of the least weasel, Mustela nivalis. *J. Mammal.* **49**: 548-550.
63. Hunsaker D., and T.C. Hahn. 1965. Vocalization of the South American tapir, Tapirus terrestris. *Anim. Behav.* **13**: 69-74.
64. Jacobs, D.S., R.M.R. Barclay, and M.H. Walker. 2007. The allometry of echolocation call frequencies of insectivorous bats: why do some species deviate from the pattern? *Oecologia* **152**: 583-594.
65. Kaltwasser, M.T. 1990. Acoustic signaling in the black rat (Rattus rattus). *J. Comp. Psychol.* **104**: 227.
66. Klinck, H., D.K. Mellinger, K. Klinck, J. Hager, L. Kindermann, and O. Boebel. 2010. Long-range underwater vocalizations of the crabeater seal (Lobodon carcinophaga).  *J. Acoust. Soc. Am.* **128**: 474-479.
67. Knowlton, A., C.W. Clark, and S. Kraus. 1991. Sounds recorded in the presence of sei whale, Balaenoptera borealis. *J. Acoust. Soc. Am.* **89**: 1968.
68. Kreiss, C.M., O. Boebel, H. Bornemann, L. Kindermann, H. Klinck, K. Klinck, ... and I. van Opzeeland. 2013. Call characteristics of high-double trill leopard seal (Hydrurga leptonyx) vocalizations from three Antarctic locations. *Polarforschung* **83**: 63-71.
69. Kudo, H. 1987. The study of vocal communication of wild mandrills in Cameroon in relation to their social structure. *Primates* **28**: 289-308.
70. Le Roux, A., M.I. Cherry, and M.B. Manser. 2009. The vocal repertoire in a solitary foraging carnivore, Cynictis penicillata, may reflect facultative sociality. *Naturwissenschaften* **96**: 575-584.
71. Leger, D.W., S.D. Berney-Key, and P.W. Sherman. 1984. Vocalizations of Belding's ground squirrels (Spermophilus beldingi). *Anim. Behav.* **32**: 753-764.
72. Lemasson, A., M.A. Mikus, C. Blois-Heulin, and T. Lodé. 2014. Vocal repertoire, individual acoustic distinctiveness, and social networks in a group of captive Asian small-clawed otters (Aonyx cinerea). *J. Mammal.* **95**: 128-139.
73. Levenson, C. 1974. Source level and bistatic target strength of the sperm whale (Physeter catodon) measured from an oceanographic aircraft. *J. Acoust. Soc. Am.* **55**: 1100.
74. Ljungblad, D.K., P.O. Thompson, and S.E. Moore. 1982. Underwater sounds recorded from migrating bowhead whales, Balaena mysticetus, in 1979. *J. Acoust. Soc. Am.* **71**: 477.
75. Lloyd, J.E. 1972. Vocalization in Marmota monax. *J.Mammal.* **53**: 214-216. doi: 10.2307/1378854
76. Long, A.M., N.P. Moore, and T.J. Hayden. 1998. Vocalizations in red deer (Cervus elaphus), sika deer (Cervus nippon), and red× sika hybrids. *J.Zool.* **244**: 123-134.
77. Macedonia, J.M. 1986. Individuality in a contact call of the ringtailed lemur (Lemur catta). *Am. J. Primatol.* **11**: 163-179.
78. Macias, S., E.C. Mora, A. Garcia, and Y. Macias. 2006. Echolocation Behavior of Brachyphylla nana (Chiroptera: Phyllostomidae) under Laboratory Conditions. *Carib. J. Sci.* **42**: 114.
79. MacKinnon, J. 1974. The behaviour and ecology of wild orang-utans (Pongo pygmaeus). *Anim. Behav.* **22**: 3-74.
80. Madsen, P., M. Wahlberg, and B. Møhl. 2002. Male sperm whale (Physeter macrocephalus) acoustics in a high-latitude habitat: implications for echolocation and communication. *Behav. Ecol.Sociobiol.* **53**: 31-41.
81. May‐Collado, L.J., I. Agnarsson, and D. Wartzok. 2007. Reexamining the relationship between body size and tonal signals frequency in whales: a comparative approach using a novel phylogeny. *Aquat. Mammal Sci.* **23**: 524-552.
82. McDonald, M.A., S.L. Mesnick, and J.A. Hildebrand. 2006. Biogeographic characterization of blue whale song worldwide: using song to identify populations. *J. Cetacean Res. Manag.* **8**: 55-65.
83. McShane, L.J., J.A. Estes, M.L. Riedman, and M.M. Staedler. 1995. Repertoire, structure, and individual variation of vocalizations in the sea otter. *J. Mammal.* **76**: 414-427.
84. Miller, J.R., and M.D. Engstrom. 2010. Stereotypic vocalizations in harvest mice (Reithrodontomys): Harmonic structure contains prominent and distinctive audible, ultrasonic, and non-linear elements. *J. Acoust. Soc. Am.* **128**: 1501.
85. Minami, M., and T. Kawamichi. 1992. Vocal repertoires and classification of the sika deer Cervus nippon. *J. Mammal. Soc. Japan* **17**: 71-94.
86. Moehlman, P.D. 1998. Behavioral patterns and communication in feral asses (Equus africanus). *Appl. Anim. Behav. Sci.* **60**: 125-169.
87. Moran, G. 1984. Vigilance behaviour and alarm calls in a captive group of meerkats, Suricata suricatta. *Z. Tierpsychol.* **65**: 228-240.
88. Moynihan, M. 1964. *Some behavior patterns of platyrrhine monkeys: I. The night monkey (Aotus trivirgatus)* (Vol. 146): Smithsonian Institution.
89. Moynihan, M. 1966. Communication in the titi monkey, Callicebus. *J. Zool.* **150**: 77-127.
90. Murrant M.N., J. Bowman, C.J. Garroway, B. Prinzen, H. Mayberry, et al. 2013. Ultrasonic Vocalizations Emitted by Flying Squirrels. *PLoS ONE* **8**: e73045. doi: 10.1371/journal.pone.0073045
91. Nair, R.V., and R.S. Lalmohan. 1975. Studies on the vocalisation of the sea cow Dugong dugon in captivity. *Indian J. Fisheries* **22**: 277-278.
92. Nelson, J.E. 1964. Vocal communication in Australian flying foxes (Pteropodidae; Megachiroptera). *Z. Tierpsychol.* **21**: 857-870.
93. Newman, J.D. 1985. Squirrel monkey communication. In *Handbook of squirrel monkey research* (pp. 99-126): Springer.
94. Norris, K.S., and W.A. Watkins. 1971. Underwater sounds of Arctocephalus philippii, the Juan Fernández fur seal. *Antarct. Res. Ser* **18**: 169-171.
95. Oleson, E.M., J. Calambokidis, W.C. Burgess, M.A. McDonald, C.A. LeDuc, and J.A. Hildebrand. 2007. Behavioral context of call production by eastern North Pacific blue whales. *Mar. Ecol. Prog. Ser.* **330**: 269-284.
96. Page, B., S.D. Goldsworthy, M.A. Hindell, and J. Mckenzie. 2002. Interspecific differences in male vocalizations of three sympatric fur seals (Arctocephalus spp.). *J. Zool.* **258**: 49-56.
97. Palacios, V., E. Font, and R. Márquez. 2007. Iberian wolf howls: Acoustic structure, individual variation, and a comparison with North American populations. *J.Mammal.* **88**: 606-613.
98. Palombit, R.A. 1992. A preliminary study of vocal communication in wild long-tailed macaques (Macaca fascicularis). I. Vocal repertoire and call emission. *Int. J. Primatol* **13**: 143-182.
99. Payne, K.B., W.R. Langbauer Jr, and E.M. Thomas. 1986. Infrasonic calls of the Asian elephant (Elephas maximus). *Behav. Ecol. Sociobiol.* **18**: 297-301.
100. Peters, G., and B.A. Tonkin-Leyhausen. 1999. Evolution of acoustic communication signals of mammals: friendly close-range vocalizations in Felidae (Carnivora). *J. Mamm. Evol.* **6**: 129-159.
101. Pola, Y.V., and C.T. Snowdon. 1975. The vocalizations of pygmy marmosets (Cebuella pygmaea). *Anim. Behav.* **23**: 826-842.
102. Policht, R., K. Tomášová, D. Holečková, and D. Frynta. 2008. The vocal repertoire in Northern white rhinoceros Ceratotherium simum cottoni as recorded in the last surviving herd. *Bioacoustics* **18**: 69-96.
103. Rasmussen, M.H., and L.A. Miller. 2002. Whistles and clicks from white-beaked dolphins, Lagenorhynchus albirostris, recorded in Faxafloi Bay, Iceland. *Aquat. Mamm.* **28**: 78-89.
104. Renouf, D., G. Galway, and L. Gaborko. 1980. Evidence for echolocation in harbour seals. *J. Mar. Biol. Assoc. UK* **60**: 1039-1042.
105. Richardson, L.W., H.A. Jacobson, R.J. Muncy, and C.J. Perkins. 1983. Acoustics of white-tailed deer (Odocoileus virginianus). *J. Mammal.* **64**: 245-252.
106. Richardson, W.J., and D.H. Thomson. 1995. *Aquatic mammals and noise*: San Diego; Toronto: Academic Press.
107. Robbins, R.L. 2000. Vocal communication in free-ranging African wild dogs (Lycaon pictus). *Behaviour* **137**: 1271-1298.
108. Roberts, L.H. 1972. Variable resonance in constant frequency bats. *J. Zool.* **166**: 337-348.
109. Robinson, J.G. 1979. An analysis of the organization of vocal communication in the titi monkey Callicebus moloch. *Z. Tierpsychol.* **49**: 381-405.
110. Robinson, J.G. 1984. Syntactic structures in the vocalizations of wedge-capped capuchin monkeys, Cebus olivaceus. *Behaviour* **90**: 46-79.
111. Rogers, T. L. 2007. Age-related differences in the acoustic characteristics of male leopard seals, Hydrurga leptonyx.  *J. Acoust. Soc. Am.* **122**: 596-605.
112. Rogers, T.L., and S.M. Brown. 1999. Acoustic observations of Arnoux's beaked whale (Berardius arnuxii) off Kemp Land, Antarctica. *Mar. Mammal. Sci.* **15**: 192-198.
113. Rogers, T.L., and D.H. Cato. 2002. Individual variation in the acoustic behaviour of the adult male leopard seal, Hydrurga leptonyx. *Behaviour* **139**: 1267-1286.
114. Rogers, T.L., D.H. Cato, and M.M. Bryden. 1995. Underwater vocal repertoire of the leopard seal (Hydrurga leptonyx) in Prydz Bay, Antarctica. *Sensory Systems of Aquatic Mammals*, 223-236.
115. Rogers, T.L., D.H. Cato, and M.M. Bryden. 1996. Behavioral significance of underwater vocalizations of captive leopard seals, Hydurga Leptonyx. *Mar. Mammal Sci.* **12**: 414-427.
116. Sanvito, S., and F. Galimberti. 2000. Bioacoustics of southern elephant seals. I. Acoustic structure of male aggressive vocalisations. *Bioacoustics* **10**: 259-285.
117. Schoeman, C.M., and D.S. Jacobs. 2003. Support for the allotonic frequency hypothesis in an insectivorous bat community. *Oecologia* **134**: 154-162.
118. Schultz, K.W., and P.J. Corkeron. 1994. Interspecific differences in whistles produced by inshore dolphins in Moreton Bay, Queensland, Australia. *Can. J. Zoolog.* **72**: 1061-1068.
119. Schusterman, R.J., R. Gentry, and J. Schmook. 1967. Underwater sound production by captive California sea lions, Zalophus californianus. *Zoologica* **52**: 21-24.
120. Sieber, O. J. 1984. Vocal communication in raccoons (Procyon lotor). *Behaviour* **90**: 80-113.
121. Simmons, J.A., S.A. Kick, and B.D. Lawrence. 1984. Echolocation and hearing in the mouse-tailed bat,Rhinopoma hardwickei: acoustic evolution of echolocation in bats. *J.Comp.Physiol. A* **154**: 347-356. doi: 10.1007/BF00605234
122. Sjare, B.L., and T.G. Smith. 1986. The vocal repertoire of white whales, Delphinapterus leucas, summering in Cunningham Inlet, Northwest Territories. *Can. J. Zoolog.* **64**: 407-415.
123. Smith, M. 1980. Behaviour of the Koala, Phascolarctos cinereus (Goldfuss), in Captivity III*. Vocalisations. *Wildlife Res.* **7**: 13-34.
124. Steiner, W.W. 1981. Species-specific differences in pure tonal whistle vocalizations of five western North Atlantic dolphin species. *Behav. Ecol. Sociobiol.* **9**: 241-246.
125. Stirling, I. 1973. Vocalization in the ringed seal (Phoca hispida). *J. Fish. Board Can.* **30**: 1592-1594.
126. Stirling, I., and D.B. Siniff. 1979. Underwater vocalizations of leopard seals (Hydrurga leptonyx) and crabeater seals (Lobodon carcinophagus) near the South Shetland Islands, Antarctica. *Can. J. Zoolog.* **57**: 1244-1248.
127. Stirling, I., and R.M. Warneke. 1971. Implications of a comparison of the airborne vocalizations and some aspects of the behaviour of the two Australian fur seals, Arctocephalus spp., on the evolution and present taxonomy of the genus. *Aust. J. Zool.* **19**: 227-241.
128. Stirling, I., W. Calvert, and H. Cleator. 1983. Underwater vocalizations as a tool for studying the distribution and relative abundance of wintering pinnipeds in the high Arctic. *Arctic* **36**: 262-274.
129. Svendsen, G.E. 1976. Vocalizations of the long-tailed weasel (Mustela frenata). *J. Mammal.* **57**: 398-399.
130. Terhune, J.M., and K. Ronald. 1973. Some hooded seal (Cystophora cristata) sounds in March. *Can. J. Zoolog.* **51**: 319-321.
131. Theis, K.R., K.M. Greene, S.R. Benson-Amram, and K.E. Holekamp. 2007. Sources of variation in the long-distance vocalizations of spotted hyenas. *Behaviour* **144**: 557-584.
132. Thomas, J.A., and I. Stirling. 1983. Geographic variation in the underwater vocalizations of Weddell seals (Leptonychotes weddelli) from Palmer Peninsula and McMurdo Sound, Antarctica. *Can. J. Zoolog.* **61**: 2203-2212.
133. Thomas, J.A., S.R. Fisher, W.E. Evans, and F.T. Awbrey. 1983. Ultrasonic vocalizations of leopard seals (Hydrurga leptonyx). *Antarct J* **17**: 186.
134. Thompson, P.O., W.C. Cummings, and S.J. Ha. 1986. Sounds, source levels, and associated behavior of humpback whales, southeast Alaska. *J. Acoust. Soc. Am.* **80**: 735.
135. Tripovich, J.S., T.L. Rogers, and J. P. Arnould. 2005. Species-specific characteristics and individual variation of the bark call produced by male Australian fur seals, Arctocephalus pusillus doriferus. *Bioacoustics* **15**: 79-96.
136. Tripovich, J.S., R. Canfield, T.L. Rogers, and J.P. Arnould. 2008. Characterization of Australian fur seal vocalizations during the breeding season. *Mar. Mammal Sci.* **24**: 913-928.
137. Tripovich, J.S., T.L. Rogers, R. Canfield, and J.P. Arnould. 2006. Individual variation in the pup attraction call produced by female Australian fur seals during early lactation.  *J. Acoust. Soc. Am.* **120**: 502-509.
138. Tripovich, J.S., I. Charrier, T.L. Rogers, R. Canfield, and J.P. Arnould. 2008. Acoustic features involved in the neighbour–stranger vocal recognition process in male Australian fur seals. *Behav. Process.* **79**: 74-80.
139. Veitl, S., S. Begall, and H. Burda. 2000. Ecological determinants of vocalisation parameters: The case of the coruro Spalacopus cyanus (Octodontidae), a fossorial social rodent. *Bioacoustics* **11**: 129-148. doi: 10.1080/09524622.2000.9753455
140. Volodina, E.V. 2000. Vocal repertoire of cheetah Acinonyx jubatus (Carnivora, Felidae) in captivity: sound structure and search for means of assessing the state of adult animals. *Entomol. Rev.* **80**: S368.
141. Watkins, W.A. 1968. The harmonic interval: fact or artifact in spectral analysis of pulse trains. Woods Hole Oceanographic Institution.
142. Watkins, W.A., and G.C. Ray. 1985. In‐air and underwater sounds of the Ross seal, Ommatophoca rossi. *J. Acoust. Soc. Am.* **77**: 1598.
143. Watkins, W.A., and W.E. Schevill. 1972. *Sound source location by arrival-times on a non-rigid three-dimensional hydrophone array.* Paper presented at the Deep Sea Research and Oceanographic Abstracts.
144. Watts, C.H.S. 1975. Vocalizations of Australian hopping mice (Rodentia: Notomys). *J. Zool.* **177**: 247-263.
145. Watts, C.H.S. 1976. Vocalizations of the plains rat Pseudomys australis Gray (Rodentia : Muridae). *Aust. J. Zool.* **24**: 95-103.
146. Wong, J., P.D. Stewart, and D.W. MacDonald. 1999. Vocal Repertoire in the European Badger (Meles meles): Structure, Context, and Function. *J. Mammal.* **80**: 570-588.
147. Zimmermann, E. 1985. Vocalizations and associated behaviours in adult slow loris (Nycticebus coucang). *Folia primatol.* **44**: 52-64.
