# Supplementary material for: Does size matter? Examining the drivers of mammalian vocalizations
Source: Evolution. 2016 Dec 13;71(2):249–60. doi: 10.1111/evo.13128 (PMC5324685; doi:10.1111/evo.13128)
Supplement: Supplementary file 2 — Supporting Information S2. A comparison of the level of support for possible explanatory models that describe the evolution of the a) minimum and b) maximum frequency in vocalisations of mammals. [file EVO-71-249-s002.pdf]

Supporting Information S2. a) A comparison of the level of support for possible explanatory models that describe the evolution of the minimum frequency in vocalisations of mammals. Excludes species for which interpolated data was used in the making of the phylogeny. The results are produced from phylogenetic generalised least squares (PGLS) analysis. T is terrestrial, S is semi-aquatic and A is aquatic.

Model	Δ*AICc*	Weighted AIcc	95% CI of slope parameter (Lower, Upper)	PGLS λ	λ 95% CI (Lower, Upper)	Effect size (r)
$\beta_0+\beta_{mass}+\beta_{environment}$	0.00	0.6424	-0.50, -0.30	0.55	0.55, 0.56	0.54
$\beta_0+\beta_{mass}+\beta_{environment}+\beta_{sociality}$	2.11	0.2237	-0.50, -0.29	0.55	0.55, 0.56	0.54
		0.0767	-0.21, 0.26 T			
$\beta_0+\beta_{mass*}\beta_{environment}$	4.25		-0.44, 0.49 S	0.53	0.53, 0.54	0.54
			-0.62, -0.22 A			
$\beta_0+\beta_{mass}$	4.84	0.0571	-0.41, -0.19	0.79	0.79, 0.79	0.40
β_0	26.35	0.0000	-	0.89	0.88, 0.89	-

b) A comparison of the level of support for possible explanatory models that describe the evolution of the maximum frequency in vocalisations of mammals. Excludes species for which interpolated data was used in the making of the phylogeny. The results are produced from phylogenetic generalised least squares (PGLS) analysis. T is terrestrial, S is semi-aquatic and A is aquatic.

Model	Δ*AICc*	Weighted AICc	95% CI of slope parameter (Lower, Upper)	PGLS λ	λ 95% CI (Lower, Upper)	Effect size (r)
$\beta_0+\beta_{mass*}\beta_{environment}$	0.00	0.7781	-0.38, -0.06 T<br>-0.46, 0.30 S<br>-0.25, 0.03 A	0.32	0.32, 0.32	0.56
$\beta_0+\beta_{mass}+\beta_{environment}$	3.11	0.1643	-0.33, -0.19	0.43	0.43, 0.43	0.49
$\beta_0+\beta_{mass}+\beta_{environment}+\beta_{sociality}$	5.21	0.0575	-0.33, -0.19	0.43	0.43, 0.44	0.49
$\beta_0+\beta_{mass}$	19.01	0.0001	-0.22, -0.06	0.71	0.70, 0.71	0.26
β_0	27.96	0.0000	-	0.78	0.78, 0.78	-
